# Cigarette Smoking and Electronic Cigarettes Use: A Meta-Analysis

**DOI:** 10.3390/ijerph13010120

**Published:** 2016-01-12

**Authors:** Meng Wang, Jian-Wei Wang, Shuang-Shuang Cao, Hui-Qin Wang, Ru-Ying Hu

**Affiliations:** 1 Zhejiang Provincial Center for Disease Control and Prevention, 3399 Binsheng Road, Hangzhou 310051, China; mwang@cdc.zj.cn; 2 Yidu Central Hospital of Weifang. 4138 Linglong Road, Qingzhou, 262500, Shandong Province, China; wangjw8711@163.com (J.-W.W.); caoss1234@163.com (S.-S.C.); 3 Department of Preventative Medicine, School of Medicine, Ningbo University, 818 Fenghua Road, Ningbo 315211, Zhejiang Province, China; wanghuiqin1990121@163.com

**Keywords:** cigarette smoking, electronic cigarette, meta-analysis

## Abstract

Increasing evidence indicates that cigarette smoking is a strong predictor of electronic cigarettes (e-cigarettes) use, particularly in adolescents, yet the effects has not be systematically reviewed and quantified. Relevant studies were retrieved by searching three databases up to June 2015. The meta-analysis results were presented as pooled odds ratios (ORs) with 95% confidence intervals (CIs) calculated by a random-effects model. Current smokers were more likely to use e-cigarette currently (OR: 14.89, 95% CI: 7.70–28.78) and the probability was greater in adolescents than in adults (39.13 *vs.* 7.51). The probability of ever e-cigarettes use was significantly increased in smokers (OR: 14.67, 95% CI: 11.04–19.49). Compared with ever smokers and adults, the probabilities were much greater in current smokers (16.10 *vs.* 9.47) and adolescents (15.19 *vs.* 14.30), respectively. Cigarette smoking increases the probability of e-cigarettes use, especially in current smokers and adolescents.

## 1. Introduction

Electronic cigarettes (e-cigarettes) are battery-operated devices that do not burn or use tobacco leaves but instead vaporize a solution the user then inhales [1]. Since they were invented in 2003, e-cigarettes have captured considerable attention with huge controversy. On the one hand, e-cigarettes are supported as safer than conventional cigarettes and effective as smoking cessation aids [2,3]. On the other hand, there are a host of concerns about the potential health problems [4,5,6] and some reports do not support their efficacy for smoking cessation [7,8]. While the issues of safety and efficacy for cessation are under heated debate, e-cigarettes have gained popularity among both adolescents and adults. During the period of 2011–2012, the lifetime e-cigarettes use prevalence in US adolescents doubled from 3.3% to 6.8%, and the similar rising trend in e-cigarettes use also emerged in adults [9,10,11]. Additionally, findings from the International Tobacco Control (ITC) Surveys covering 10 countries further indicate that the use of e-cigarettes has increased substantially globally [12].

Various potential factors have been reported to be associated with e-cigarettes use, including gender [13,14], socio-economic status [14,15], parental or friends smoking [14,15,16], and e-cigarettes harm perception [14,16] and, most importantly, the cigarette smoking status. Although e-cigarettes may be used as a gateway to cigarette smoking by non-smokers, considerable studies have revealed that most e-cigarettes users are or were cigarette smokers in both adolescents and adults [10,13,17,18]. The relevant literature is increasing rapidly, but to date the effect of cigarette smoking on e-cigarettes use has not be systematically reviewed and quantified. Therefore, we retrieve the available literature and conduct a meta-analysis to provide the summary estimates of the effects.

## 2. Materials and Methods

### 2.1. Literature Search Strategy

We performed comprehensive searches of three databases (PubMed, Springer Link, Elsevier) from 2003 to June, 2015 to identify epidemiological studies on the association between cigarette smoking and e-cigarettes use. There were no language restrictions in the procedure of literature search, but only English papers were included in the meta-analysis. Ever use of e-cigarette was defined as “I have tried one” or “I have tried one, but do not smoke one in the past month”; current use of e-cigarette was defined as “I have smoked one in the past month”. Ever and current cigarette smoking followed the above definitions. Detailed definitions were shown in Table A1 in the Appendix. The main search terms included “electronic cigarette”, “e-cigarette”, “electronic nicotine delivery systems”, “vaping”, “vaper”, “vapor”, “smoking”, “cigarette smoking”, “tobacco smoking”, and “tobacco use”. Reference lists of retrieved literature were also screened. The current study was carried out followed the Meta-analysis of Observational Studies in Epidemiology (MOOSE) guidelines [19].

### 2.2. Eligibility Criteria and Data Extraction

We selected studies that: (1) reported the association between cigarette smoking and e-cigarettes use (2) provided the odds ratios (ORs) with 95% confidence intervals (CIs) for highest *vs.* lowest status of cigarette smoking or raw data to calculate these. Eligibility of studies was assessed and relevant information was extracted from each eligible study independently by two authors. The information included author”s name, year of publication, data source, location where the study conducted, sample size, study type, smoking status classification, and variables adjusted. The quality of each eligible study was assessed by the 9-star Newcastle-Ottawa Scale [20], a validated technique for assessing the quality of observational studies.

### 2.3. Statistical Analysis

A random effects model was used to calculate the pooled ORs with 95% CIs for cigarette smoking. Heterogeneity between studies was assessed using Q-test and the I^2^ statistic [21]. To explore the possible sources of heterogeneity, subgroup analyses were conducted based on cigarette smoking (ever and current smoking) and age group [adolescents (mean age < 18 years) and adults (mean age ≥ 18 years)], respectively. However, owing to the lack of data, analysis on the effect of ever smoking on current e-cigarettes use was not feasible. To test robustness of the current meta-analysis results, sensitivity analyses were also performed with excluding outliers. Publication bias was assessed by Egger′s regression asymmetry test [22] and Begg′s rank correlation test [23] (*p* values < 0.05 were considered statistically significant). All the statistical analyses were conducted with STATA Version 11 software (StataCorp LP: College Station, TX, USA).

## 3. Results

### 3.1. Overview of Included Studies

**Figure 1:**
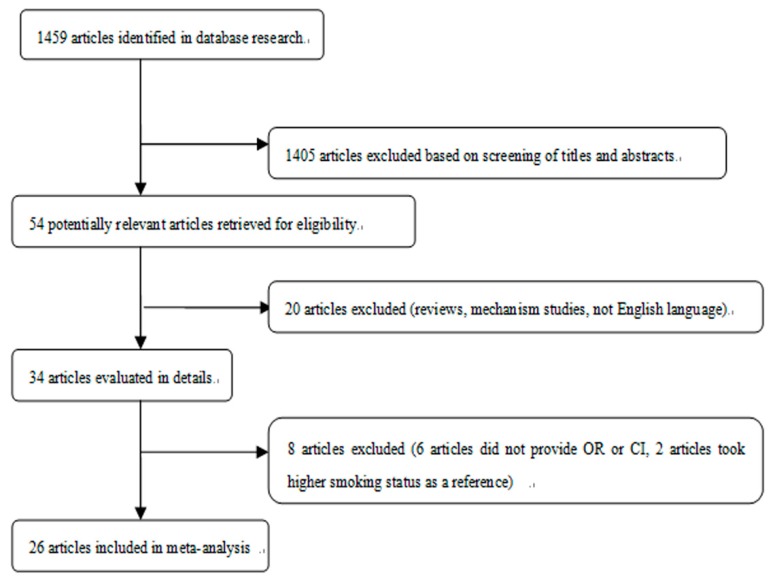
Study selection process.

Figure 1 showed the detailed procedures of study selection for this meta-analysis. Among the 54 potentially eligible studies, 20 articles were excluded because they were reviews, studies on mechanism or published without English language. A further eight studies not providing or providing unsuitable ORs and CIs for meta-analysis were also excluded. The detailed information of studies was shown in the Appendix, Table A2. Briefly, we identified 26 studies published between 2011 and 2015 for this meta-analysis. Most of studies were from the USA and Europe. The majority of included studies adjusted potential confounders for the final estimates, except for only three studies [11,24,25]. In the publication of Barnett *et al.* [26], results were respectively shown for middle and high school students, and were treated as two independent studies. As with the publication of Barnett *et al.*, different results in the publications of Camenga *et al.* [27], King *et al.* [28], Ramo *et al.* [25] and Moore *et al.* [24] were also included in the current meta-analysis as independent studies. The quality score of studies ranged from 3 stars to 8 stars according to the 9-star Newcastle-Ottawa Scale.

### 3.2. Meta-Analysis of Association between Current Smoking and Current E-Cigarettes Use

**Figure 2:**
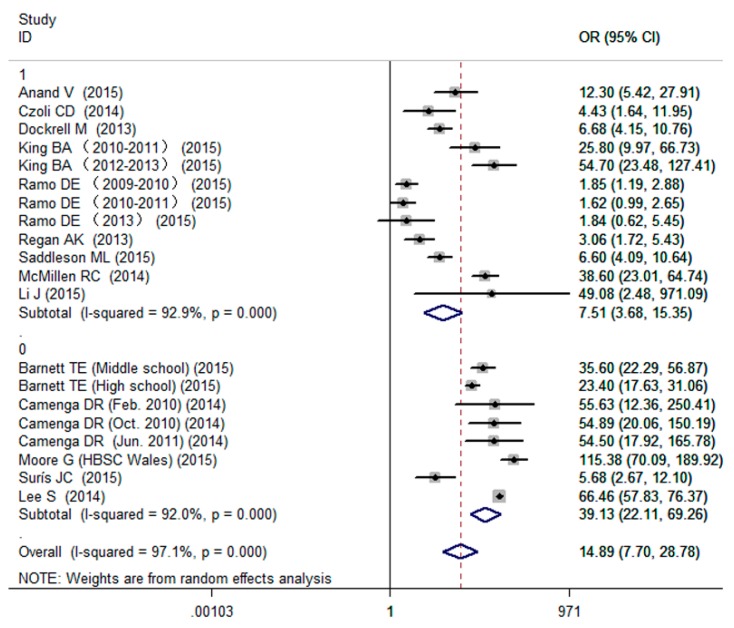
Effects of current smoking on current e-cigarettes use among adolescents and adults (0: adolescent 1: adult).

The pooled analysis found that individuals with a status of current smoking had a great probability of current e-cigarettes use (OR = 14.89, 95% CI: 7.70–28.78; Figure 2), although the formal test for heterogeneity gave a significant result (I^2^ = 97.1%). Subgroup analysis based on age group was conducted and indicated that the effects of current smoking on current e-cigarettes use were greater in adolescents (OR = 39.13, 95% CI: 22.11–69.26; Figure 2) than in adults (OR = 7.51, 95% CI: 3.68–15.35; Figure 2). After excluding the outlier, the sensitivity analysis result of pooled OR was 13.27 (95% CI: 6.71–26.24).

### 3.3. Meta-Analysis of Association between Cigarette Smoking and Ever E-Cigarettes Use

**Figure 3:**
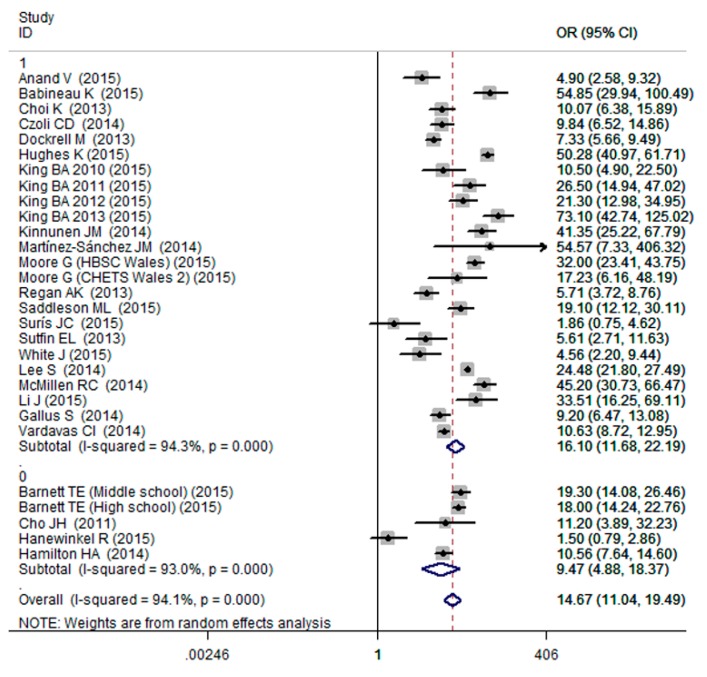
Effects of ever and current cigarette smoking on ever e-cigarettes use (0: ever smoking; 1: current smoking).

**Figure 4:**
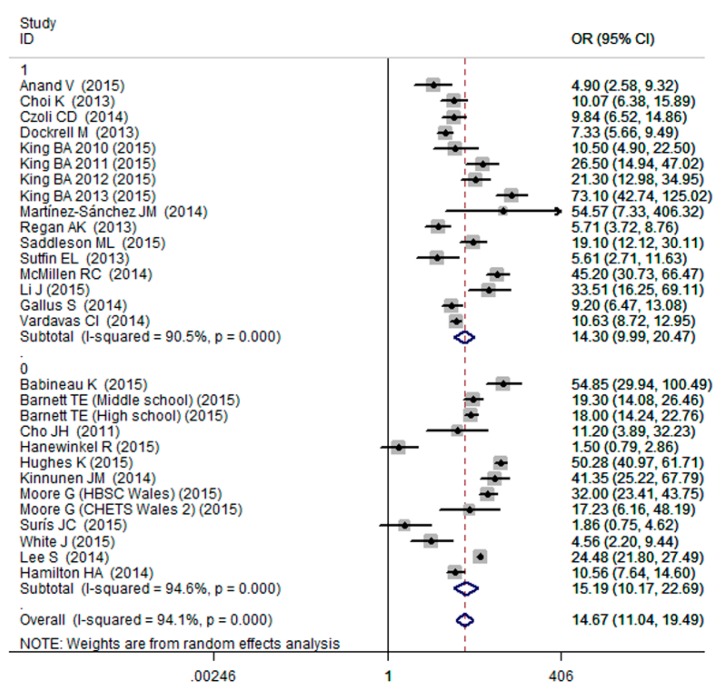
Effects of cigarette smoking on ever e-cigarettes use among adolescents and adults (0: adolescent; 1: adult).

The results of meta-analysis showed that cigarette smoking was associated with increased probability of ever e-cigarettes use (OR = 14.67, 95% CI: 11.04–19.49; Figure 3), with a high evidence of between-study heterogeneity (I^2^ = 94.1%; Figure 3). To further explore the possible sources of heterogeneity, subgroup analyses were conducted based on cigarette smoking status and age group. When subgroup analysis was conducted based on cigarette smoking status, we observed the pooled ORs were 16.10 (95% CI: 11.68–22.19; Figure 3), 9.47 (95% CI: 4.88–18.37; Figure 3) for the current smoking and ever smoking, respectively. When subgroup analysis was conducted based on age group, the pooled ORs were 14.30 (95% CI: 9.99–20.47; Figure 4), 15.19 (95% CI: 10.17–22.69; Figure 4) for the adults and adolescents, respectively. After excluding the outlier, the sensitivity analysis result of pooled OR was 13.84 (95% CI: 10.41–18.40).

### 3.4. Publication Bias

No evidence of publication bias was detected in the analysis of the associations between smoking and either current e-cigarettes use or ever e-cigarettes use (data not shown).

## 4. Discussion

This paper presents findings from the first meta-analysis to synthesize summary estimates of the effect of cigarette smoking on e-cigarettes use. Overall, our analysis results provided evidence that cigarette smoking, especially current smoking, was associated with great probability of current and ever e-cigarettes use. Recently, Wu *et al.* conducted a review on e-cigarettes prevalence and correlates of use, which came to the consistent conclusion that cigarette smoking was the only common correlate of e-cigarettes use in both adolescents and adults [18]. Furthermore, in the subgroup analyses, our observations suggested that the effect of cigarette smoking on e-cigarettes use was greater in adolescents than in adults. Similarly, Vardavas *et al.* performed a second analysis of Eurobarometer data of 26,566 youth and adults from 27 countries, with the finding that ever e-cigarettes use was more likely among younger current smokers [29]. Considering the facts that the safety information and the cessation properties of e-cigarettes are sparse and inconsistent, more solid public health evidence on e-cigarettes” long-term safety and smoking cessation efficacy are urgently needed.

Although the mechanisms by which cigarette smoking increased the probability of e-cigarettes use, especially in adolescents, were complicated, we advocated that smokers tend to accept e-cigarettes. First, previous study indicated that being a smoker was the strongest predictor of the willingness to try an e-cigarette [30]. Second, cigarette smokers were more likely to be susceptible to the e-cigarettes ads than non-smokers. Smith *et al.* conducted a web-based survey and the results indicated that exposure to e-cigarettes ads might enhance interest in e-cigarettes trial, particularly among cigarette smokers [31]. Since e-cigarettes were mostly advertised through the Internet, a study by Regan *et al.* further suggested that younger people were more exposed to e-cigarettes ads than older adults [11], which provided a possible interpretation of the greater effect of cigarette smoking on e-cigarettes use among adolescents. Additionally, according to previous literature, sensation seeking and the willingness to try new also contributed to the adolescent smokers” susceptibility to e-cigarettes use [18,30,32]. Third, e-cigarettes users generally perceived e-cigarettes were healthier than conventional cigarettes [7,33,34] and studies suggested that those perceiving e-cigarettes as less harmful than cigarettes were more likely to use them [7,16,29,35]. To current and ever smokers, there was no exception. Evidence indicated that majority of cigarette smokers believed that e-cigarettes were safer than conventional cigarettes with the potential benefits for aiding to quit smoking [34,36,37].

The results from our meta-analysis study were subject to several limitations. One limitation was that high levels of heterogeneity were observed in the summary estimates, suggesting unexplained factors for variation still exist. As for the possible sources of heterogeneity, we attempted to speculate from the following aspects. First, most studies we assessed relied on the self-reported e-cigarettes use, which might underestimate the observed effects and contribute to the high levels of heterogeneity. Second, smaller estimates with narrow confidence interval were seen in studies conducted before 2013, while the effect seemed to be more pronounced with larger estimates in recent years of 2013 and 2014. Considering the e-cigarettes were newly sprouted things, which promoted and spread as aids for smoking cessation among smokers, we speculated that the year of the study explained part of heterogeneity. Third, different cultures, regulations on e-cigarettes, and source populations between the studies might be also responsible for the heterogeneity. Another limitation of our study was that most studies included in the current meta-analysis are cross-sectional studies, and we cannot identify the causality and rule out the residual confounding in some or all of the studies. Besides, most studies included in the meta-analysis defined “past 30-days use” as current use, which may include those who simply tried once in the last month.

## 5. Conclusions

In summary, this meta-analysis study indicates that cigarette smoking increases the probability of e-cigarettes use, especially in current smokers and adolescents. Given that our study is mostly based on cross-sectional studies, more evidence from longitudinal researches is needed.

## Appendix

**Table A1:** Definition of the e-cigarettes and cigarettes smoking status in studies included in the meta-analysis.

Citation	The Definition of E-Cigarettes Use Status	The Definition of Cigarette Smoking Status
Anand V, *et al.* 2015 [38]	The life time use: have ever tried an e-cigarette. Current use (Occasional use: at least one use occasion during the past month. Regular use: at least 10 use occasions during the past month. Daily use)	1. The life time use: have ever tried a cigarette. 2. Current use (Occasional use: at least one use occasion during the past month. Regular use: at least 10 use occasions during the past month Daily use)
Babineau K, *et al.* 2015 [39]	Ever Users (those who had tried e-cigarettes)	1. Ever smoker: those who have tried smoking once or twice or have quit. 2. Current smoker: those who smoke at least once a month
Barnett TE, *et al.* 2015 [26]	Ever user: “have you ever tried, even once” (yes). Current user: “during the past 30 days, have you used an e-cigarette”	1. “Ever cigarette use” was determined using the following question: Have you ever tried cigarette smoking, even one or two puffs? If the respondents answered yes they were categorized as an “ever cigarette user.” 2. “Current cigarette use” was determined using the following question: During the past 30 days, on how many days did you smoke cigarettes? If the respondents answered 1 or more, they were categorized as a “current cigarette user.”
Camenga DR, *et al.* 2014 [27]	Past-30 day use of ENDS was measured by the response to the question “In the PAST 30 DAYS, have you used any of the following tobacco products?” by selecting the option “E-cigarettes (An electronic cigarette that is filled with liquid nicotine)”.	Current smokers: In the PAST 30 DAYS, have you used tobacco? “Yes”
Cho JH, *et al.* 2011 [40]	“Have you ever smoked an e-cigarette, even one or two puffs?” If they answered in the affirmative, they were classified as having had the experience of e-cigarette use.	Cigarette smoking experience: Have you ever smoked an e-cigarette, even one or two puffs?” If they answered in the affirmative, they were classified as having had the experience of e-cigarette use.
Choi K, *et al.* 2013 [16]	We asked those who were aware of e-cigarettes whether they had ever used e-cigarettes (yes).	1. Former smoker: those who smoked 100 or over cigarettes but had not smoked in the past 30 days. 2. Current smoker: those who smoked 100 or over cigarettes and had smoked in the past 30 days.
Czoli CD, *et al.* 2014 [41]	e-cigarette “ever-users” had tried an e-cigarette, but had not smoked one in the last 30 days; and “current users” of e-cigarettes had tried an e-cigarette and had smoked one in the last 30 days.	1. Former smokers: who had smoked 100 cigarettes in their lifetime but had not smoked in the last 30 days. 2. Current smokers were defined as those who had smoked 100 cigarettes in their lifetime and had smoked in the last 30 days;
Dockrell M, *et al.* 2013 [36]	Ever use: I have tried e-cigarettes in the past 12 months but do not currently smoke them; I have tried e-cigarettes longer than 12 months ago but do not currently smoke them Current use: I currently smoke e-cigarettes.	Ex-smoker: I used to smoke but I have given up now. Occasional smoker: I smoke but I don”t smoke every day; Daily smoker: I smoke every day.
Hanewinkel R, *et al.* 2015 [42]	Lifetime use of e-cigarettes was assessed by asking “Have you ever used an electronic cigarette?” (yes).	Ever smoked a conventional cigarette: Students having smoked at least a few puffs were considered as ever-smokers
Hughes K, *et al.* 2015 [43]	The question on e-cigarette access asked students “have you ever tried or purchased e-cigarettes”.	1. Ex-smokers were identified through the option “I used to smoke but have given up” 2. Those smoking less than 5 a day were categorized as light regular smokers and those smoking at higher levels as heavy regular smokers.
King BA, *et al.* 2015 [28]	Ever Use: ever use of e-cigarettes was assessed using the question, “Have you ever tried any of the following products, even just one time”? Respondents who selected “electronic cigarettes or e-cigarettes” were considered to be ever e-cigarette users. Current Use: current use of e-cigarettes was assessed by the question, “In the past 30 days, which of the following products have you used at least once”? Respondents who selected “electronic cigarettes or e-cigarettes” were considered to be current e-cigarette users.	1. Former smokers were respondents who smoked 100 or over cigarettes in their lifetime and reported smoking “not at all” at the time of survey. Current smokers were defined as respondents who smoked 100 or over cigarettes in their lifetime and reported smoking “everyday” or “some days” at the time of survey.
Kinnunen JM, *et al.*, 2014 [15]	“Have you ever tried electronic cigarettes?” (yes)	Experimenters (tried but did not smoke daily), and daily smokers (reported daily smoking and smoked >50 cigarettes in lifetime).
Martínez-Sánchez JM, *et al.* 2014 [44]	“Have you ever used e-cigarettes?” The answers to this question were: “yes, currently”; “yes, in the past”; “I have only experimented with e-cigarettes”	1. Former smokers as participant who did not smoke cigarettes at the moment of the survey but had smoked cigarettes in the past. 2. Current smokers as participants who smoked cigarettes either daily (at least one cigarette/day) or occasionally (less than one cigarette/day) at the moment of the survey.
Moore G, *et al.* 2015 [24]	In CHETS Wales 2, children were asked if they had ever used an e-cigarette, with response options of: “no”; “yes, once”; or “yes more than once”. In HBSC Wales, young people were asked if they had ever used an e-cigarette, with response options of: “I have never used or tried e-cigarettes”; “I have used e-cigarettes on a few occasions (1–5 times)”; or “I regularly use e-cigarettes (at least once a month)”.	Lifetime smoking was measured in CHETS Wales 2 by asking children whether they ever smoked tobacco, with response options of “yes” or “no”. In the HBSC Wales survey, respondents were asked “On how many days (if any) have you smoked cigarettes?”, with seven response options: “Never”; “1–2 days”; “3–5 days”; “6–9 days”; “10–19 days”; “20–29 days”; “30 days or more”. Respondents who report “never” smoking cigarettes are compared to the other response options to assess lifetime prevalence. In both surveys, current smoking was assessed by asking “How often do you smoke tobacco at present?” with response options of “every day”, “at least once a week, but not every day”, “less than once a week”, and “I do not smoke”.
Ramo DE, *et al.* 2015 [25]	Participants in all three studies were asked the same question: “In the past month, have you used any tobacco products other than cigarettes?” and given ten answer choices, including “e-cigarette/electronic cigarettes” and “other”; any “other” answers that included electronic cigarettes were recoded as such.	Daily smoker: smoked every day
Regan AK, *et al.* 2013 [11]	“Have you ever tried any of the following products, even just one time, including e-cigarette. “In the past 30 days, which of the following products have you used at least once”, including e-cigarette.	1. Former smokers had smoked 100 or over cigarettes in their lifetime but currently do not smoke at all. 2. Current smokers were defined as adults who reported smoking 100 or over cigarettes in their lifetime and currently smoke everyday or some days.
Saddleson ML, *et al.* 2015 [45]	E-cigarette ever use assessed by “ Have you ever tried or experimented with an e-cigarette, even one or two puffs? ” Those who responded “yes” were classified as ever users. Current use included use one or more days in the previous 30 days.	1. Former smokers (smoked≥100 cigarettes in lifetime, and have smoked 0 out of the past 30 days); 2. Experimenters (have ever tried a cigarette, have smoked < 100 cigarettes in lifetime, and have smoked 0 of the past 30 days); 3. Current smokers (have smoked at least 1 day out of the past 30). For the multivariable analyses, smoking status was collapsed into three categories (never smokers, experimenters and ever [current and former smokers]), due to few former smokers in our sample (*n* = 17).
Surís JC, *et al.* 2015 [46]	Experimenter: only once, user: several time or regularly	A current smoker was defined as smoking at least weekly.
Sutfin EL, *et al.* 2013 [47]	Ever e-cigarette users were characterized as those who responded yes. Current e-cigarette users were a subset of ever users who reported smoking an e-cigarette in the past month.	1. Former smoker or experimenter (smoked a whole cigarette in lifetime, but not in the past 30 days); 2. Current nondaily (smoked on between 1 and 29 of the past 30 days); 3. Current daily smoker (smoked on all of the past 30 days)
White J, *et al.* 2015 [48]	“Have you ever tried electronic cigarettes?” Those who answered “yes” were classified as “e-cigarette ever-users.”	1. Ex-smokers had smoked a cigarette but no longer smoked; 2. Infrequent smokers smoked less often than once a month; 3. Current smokers were those who reported smoking at least once a month or more often.
Lee S, *et al.* 2014 [49]	E-cigarette use questions were: “Have you ever used e-cigarettes?” (yes) and “Have you used e-cigarettes in the past 30 days?” (yes).	1. Former smoker was defined as a respondent who had ever smoked one puff, but had not smoked in the past 30 days. Current smoker: at least one day smoked, even one puff, in the past 30 days.
McMillen RC, *et al.* 2014 [50]	Ever use; “Have you tried Electronic Cigarettes or E-cigarettes, even just one time?” Current use: Respondents who reported every day or some days.	Respondents were asked, “Have you smoked at least 100 cigarettes in your entire life?” Respondents who reported that they had were then asked, “Do you now smoke cigarettes every day, some days, or not at all?” Respondents who reported that they have smoked at least 100 cigarettes and now smoke every day or some days were categorized as current smokers, while those who reported not at all were categorized as former smokers.
Li J, *et al.* 2015 [51]	Ever use: “Have you ever tried an electronic cigarette?” (yes) Current use: monthly use	Former smoker: Have you ever tried a cigarette (yes); current smoker: at least monthly use
Gallus S, *et al.* 2014 [52]	Ever smoker: have heard and have tried e-cigarettes.	Ever smokers (current and ex-smokers) were participants who had smoked 100 or more cigarettes in their lifetime. 1. Ex-smokers were participants who had quit smoking since at least 1 year; 2. Current smokers were individuals continuing smoking or having stopped since less than 1 year.
Hamilton HA, *et al.* 2014 [53]	“Have you ever smoked at least one puff from an electronic cigarette?” (yes)	(a)“never had a cigarette, not even one puff, in my life”; (b) “smoked from a few puffs to a whole cigarette in my life”; (c) “only 2–3 cigarettes in my life”; (d) “more than 3, but fewer than 100 cigarettes in my life”; (e) “100 or more cigarettes in my life, but none in the last month”; (f) “100 or more cigarettes in my life and some during the last month, but not every day”; and (g) “100 or more cigarettes in my life and at least 1 cigarette every day during the last month.” For purposes of this analysis, a dichotomous measure was constructed by combining Categories 2 through 7 to reflect ever smoked at least a puff of a cigarette (coded 1) *versus* never smoked (coded 0).
Vardavas CI, *et al.* 2014 [29]	Ever use of an e-cigarette was self-reported and was assessed with the question “Have you ever tried (electronic cigarettes)?” Responses of “regularly”, “occasionally”, or “tried it once or twice” were categorized as having ever tried an e-cigarette.	Smokers: self-reported of current smoke status and chose the option of current smoker.

**Table A2:** Information of the studies included in the mata-analysis.

Citation	Data Source	Location	Sample Size	Study Type	Smoking Status Classification	Variables Adjusted
Anand V, *et al.* 2015 [38]	Survey at school of military paramedical personnel	France	200 students and instructors aged 18 years or over	Cross-sectional	Tobacco current use (yes or no)	Gender and age
Babineau K, *et al.* 2015 [39]	Survey at secondary schools	Ireland	821 young people aged 16–17 years	Cross-sectional	Current tobacco user (yes or no)	Gender, school-level, socioeconomic status, birth region
Barnett TE, *et al.* 2015 [26]	2013 Florida Youth Tobacco Survey (FYTS)	Florida	6440 middle and 6175 high school students	Cross-sectional	Ever used cigarettes (yes or no); Currently use cigarettes (yes or no)	Gender, race, grade level, other tobacco use
Camenga DR, *et al.* 2014 [27]	Survey at two suburban high schools, 2010–2011	Connecticut & New York	Wave 1–1719 Wave 2–1702 Wave 3–1345, students in grades 9–12	Cross-sectional	Non-smoker; Current smokers	Study wave, school region, grade, race, gender
Cho JH, *et al.* 2011 [40]	2008 Health Promotion Fund Project	Korea	4341 students	Cross-sectional	Cigarette smoking experience (never or ever)	Gender, level of school, cigarette smoking family, propensity to be easily affected by friends, school life
Choi K, *et al.* 2013 [16]	Minnesota Adolescent Community Cohort	Minnesota	2624 adults aged 20–28 years	Cross-sectional from a cohort study	Never established smoker; Former smoker; Current smoker	Age, gender, race, education, peer smoking, perceptions of electronic cigarettes
Czoli CD, *et al.* 2014 [41]	Survey at an online panel of Canadians	Canada	1188 youth and young adults aged 16–30 years	Cross-sectional	Non-smoker; Former smoker; Current smokers	Age, gender, race, education
Dockrell M, *et al.* 2013 [36]	Population surveys in 2010 & 2012	Great Britain	2010–12,597 (2297 smokers); 2012–12,432 (2093 smokers)	Cross-sectional	Ex-smoker; Occasional smoker; Daily smoker	Age, gender, social grade
Hanewinkel R, *et al.* 2015 [42]	Survey at 45 public secondary schools	Germany	2693 adolescents aged 11–15 years	Cross-sectional from a cohort study	Ever smoked a conventional cigarette (yes or no)	Gender, age, sensation seeking, migration background, family affluence, friend, sibling, parents smoking, at a gymnasium, experimental condition
Hughes K, *et al.* 2015 [43]	5th iteration of the Trading Standards North West Alcohol and Tobacco Survey	North West England	16,193 school students aged 14–17 years	Cross-sectional	Never smoked; Tried but didn't like it; Ex-smoker; Smoke when drinking; Regular light smoker; Regular heavy smoker	Gender, age, deprivation parent/guardian smokes drinking status
King BA, *et al.* 2015 [28]	2010–2013 HealthStyles Survey	USA	2010–2505 2011–4050 2012–4170 2013–4033 Age: over 18 years	Cross-sectional	Never smoker; Former smoker; Current smoker	Gender, age, race, education, household income, region
Kinnunen JM, *et al.*, 2014 [15]	Adolescent Health and Lifestyle Survey	Finland	3535 adolescents aged 12–18 years	Cross-sectional	Never; Experimenter; Daily smoker	Age, gender, substance (snus / waterpipe) use, parents” smoking, seen e-cigarettes ads, statement ‘smoking is for loser”, school level, school performance, family structure, father and mother”s work situation.
Martínez-Sánchez JM, *et al.* 2014 [44]	Determinants of Cotinine phase 3 project (dCOT3)	Barcelona	736 adults aged 16 years or over	Cross-sectional from a cohort study	Never smoker; Former smoker; Current smoker	Gender, age, educational level
Moore G, *et al.* 2015 [24]	Child Exposure to Tobacco Smoke (CHETS) survey (‘Wales 2”); Welsh Health Behaviour in School-aged Children (HBSC) Survey (‘HBSC Wales”)	Wales	10,656 students aged 10–16 years	Cross-sectional	CHETS Wales 2: Ever smoked cigarettes (yes or no) Current tobacco use (yes or no) HBSC Wales: Ever smoked cigarettes (yes or no) ; Frequency of current tobacco use: I do not smoke; Less than once a week; At least once a week (but not every day); Every day	None
Ramo DE, *et al.* 2015 [25]	Online survey	USA	2661 adults aged 18–25 years	Cross-sectional	Daily smoker (yes or no)	None
Regan AK, *et al.* 2013 [11]	ConsumerStyles survey 2010	USA	10,328 adults aged 18 years or over	Cross-sectional	Never smoker; Former smoker; Current smoker	None
Saddleson ML, *et al.* 2015 [45]	Internet survey	Upstate New York	1437 college students aged 18–23 years	Cross-sectional	Never-smoker; Experimenter; Ever smoker	Gender, race, age, institution, school ability, other substance use, such as marijuana, alcohol, and belief that e-cigarettes are less harmful than tobacco cigarettes
Surís JC, *et al.* 2015 [46]	ado @ internet.ch study	Switzerland	621 youths with mean age of 16.2 years	Cross-sectional from a longitudinal study	Current smoker (yes or no)	Mean age, gender, academic situation, substance use (alcohol, cannabis) and substance use (alcohol, cannabis) at baseline aged 14
Sutfin EL, *et al.* 2013 [47]	Web-based survey at 8 North Carolina universities	North Carolina	4444 students with the average age of 20.5 years	Cross-sectional	Never smoked; Former smoker; Current nondaily smoker; Current daily smoker	Age, gender, race, greek off/on campus residence, sensation seeking, other substance use (hookah, binge drinking, marijuana, illegal drug), e-cigarette harm perception
White J, *et al.* 2015 [48]	Youth Insights Survey (YIS) 2014	New Zealand	2919 students aged 14–15 years	Cross-sectional	Nonsusceptible never-smoker; Susceptible never-smoker; Ex-smoker; Infrequent smoker; Current smoker	Gender, race, school decile status, weekly income, parental smoking status, close friends smoking status, past month use of other tobacco products, marijuana, ever binge drinker.
Lee S, *et al.* 2014 [49]	2011 Korean Youth Risk Behavior Web-based Survey	Korea	75,643 students aged 13–18 years	Cross-sectional	Never smoker; Former smoker; Current smoker	Gender, location, grade, weekly allowance, attempted to quit, smoking prevention program
McMillen RC, *et al.* 2014 [50]	Computer-assisted telephone survey 2013	USA	3245 adults aged 18 years or over	Cross-sectional	Never-smokers; Former smokers; Nondaily smokers; Daily smokers	Region, race, age, gender, education
Li J, *et al.* 2015 [51]	2014 Health and Lifestyles Survey (HLS)	New Zealand	2594 adults aged 15 years or over	Cross-sectional	Never smokers; Ex-smokers; Current smokers	Gender, ethnicity, age, neighbourhood deprivation
Gallus S, *et al.* 2014 [52]	An Italy national survey on smoking	Italy	3000 individuals aged 15 years or over	Cross-sectional	Never smokers; Ex-smokers; Current smokers	Gender, age, level of education, and geographic area.
Hamilton HA, *et al.* 2014 [53]	2013 Ontario Student Drug Use and Health Survey Canada (OSDUHS)	Canada	2892 students aged 19 or younger	Cross-sectional	Tobacco use, lifetime (yes or no)	Gender, grade, race, urbanicity
Vardavas CI, *et al.* 2014 [29]	Special Eurobarometer 385 (77.1)	27 countries in the European Union	26,566 youth and adults aged 15 years or over	Cross-sectional	Current smoking status: Non-smoker and smoker	Perceived harmfulness of e-cigarettes, residence, EU region, gender, age, self-reported difficulty in paying bills
